# Infectious Dermatitis in Companion Animals: A One Health Perspective

**DOI:** 10.3390/ani16152284

**Published:** 2026-07-23

**Authors:** Lola Cellier, Helena Guimarães, Ana Cardoso, Carla Campos, Patrícia Barradas, Carla Miranda

**Affiliations:** 1 University Institute of Health Sciences, Cooperativa de Ensino Superior Politécnico e Universitário (IUCS-CESPU), Avenida Central de Gandra 1317, 4585-116 Paredes, Portugal; a30486@alunos.cespu.pt (L.C.); helena.guimaraes@iucs.cespu.pt (H.G.); ana.cardoso@cespu.pt (A.C.); patricia.barradas@iucs.cespu.pt (P.B.); 2 Instituto Português de Oncologia do Porto Francisco Gentil, Rua Dr. António Bernardino de Almeida, 4200-072 Porto, Portugal; carla.campos@ipoporto.min-saude.pt; 3 Escola Superior de Saúde, Instituto Politécnico do Porto, Rua Dr. António Bernardino de Almeida, 4200-072 Porto, Portugal; 4 UCIBIO—Applied Molecular Biosciences Unit, University Institute of Health Sciences (1H-TOXRUN, IUCS-CESPU), Avenida Central de Gandra 1317, 4585-116 Paredes, Portugal; 5 Associate Laboratory i4HB—Institute of Health and Bioeconomy, University Institute of Health Sciences, CESPU, Avenida Central de Gandra 1317, 4585-116 Paredes, Portugal; 6 LAQV-REQUIMTE—Associated Laboratory for Green Chemistry, University NOVA of Lisbon, 1099-085 Caparica, Portugal

**Keywords:** antimicrobial resistance, dermatitis, pets, Portugal, public health

## Abstract

Infectious dermatitis is a common condition in companion animals and is frequently associated with opportunistic bacterial pathogens, particularly staphylococci. The emergence of antimicrobial resistance among these pathogens represents a growing challenge for veterinary medicine and public health. This study aimed to assess the presence of *Staphylococcus* species, dermatophytes, and ectoparasites in companion animals presenting clinical signs of dermatitis in Northern Portugal, and to characterize the antimicrobial resistance profiles of the isolated staphylococci. A total of 58 infectious dermatitis cases yielded 71 dermatological samples from dogs and cats between September 2025 and January 2026. *Staphylococcus* isolates were identified by MALDI-TOF MS and analyzed for phenotypic antimicrobial susceptibility and resistance genes by PCR. Twenty-nine *Staphylococcus* isolates were identified, with *S. pseudintermedius* being the predominant species (66%). Resistance to at least one antimicrobial agent was observed in 65% of isolates, while multidrug resistance was detected in 7%. The highest resistance rates were observed for penicillin (62%), tetracycline (24%), and oxacillin (21%). The most prevalent resistance genes were *tet*M (86%), *bla*Z (86%), and *mec*C (83%). No dermatophytes were detected, and two ectoparasite-positive (*Demodex* spp. and *Otodectes* spp.) cases were identified. These findings highlight the presence of antibiotic-resistant *S. pseudintermedius* in companion animal dermatitis and reinforce the importance of early antimicrobial susceptibility testing and continuous surveillance of antimicrobial resistance in veterinary dermatology.

## Simple Summary

Skin infections are among the most common health problems affecting dogs and cats, often leading to pruritus, erythema, alopecia, discomfort, and reduced quality of life. These infections are frequently associated with bacteria that are part of the normal skin microbiota but can cause disease when the skin barrier is damaged or host immunity is impaired. This study investigated the microorganisms associated with skin infections in companion animals from Northern Portugal and evaluated their resistance to commonly used antibiotics. *Staphylococcus pseudintermedius* was the most frequently identified bacterium, particularly in dogs, confirming its major role in skin disease. Most (65%) bacterial isolates showed resistance to ≥1 antibiotic, with the highest resistance observed for penicillin (62%), followed by tetracycline (24%). Several isolates also harbored antibiotic resistance genes associated with resistance, including some that appeared phenotypically susceptible, highlighting the complexity of resistance detection. No fungal skin pathogens were identified, and only a small number of ectoparasite infestations were detected. These findings underscore the importance of accurate laboratory diagnosis and antibiotic susceptibility testing prior to treatment. Improved monitoring of skin pathogens and responsible antibiotic use are essential to safeguard animal health and mitigate the spread of antibiotic resistance among animals, humans, and the environment.

## 1. Introduction

Infectious dermatitis represents one of the most frequently encountered dermatological disorders in companion animals, particularly dogs and cats. This condition is characterized by inflammation of the skin caused by opportunistic pathogens such as *Staphylococcus pseudintermedius* and *Malassezia pachydermatis*, as well as dermatophytes and ectoparasites. These microorganisms can colonize healthy skin and ears but may trigger clinical disease under favorable conditions. Clinical signs include erythema, pruritus, flaking, and other cutaneous abnormalities, often linked to allergies, immune dysfunction, or environmental factors [1,2].

Despite its high prevalence, infectious dermatitis in pets is often underdiagnosed due to overlapping clinical signs with allergic or immune-mediated skin diseases. This diagnostic challenge frequently leads to empirical antimicrobial therapy, contributing to a wider problem in veterinary and public health [3]. A major concern in the management of infectious dermatitis is the emergence of antimicrobial resistance (AMR). In veterinary medicine, systemic antibiotics such as amoxicillin–clavulanic acid, cephalosporins, and fluoroquinolones are commonly prescribed for skin infections [1]. However, their frequent and sometimes inappropriate use has been strongly associated with the emergence of resistant and multidrug-resistant (MDR, resistant to three or more classes of antimicrobials) pathogens, including methicillin-resistant *Staphylococcus pseudintermedius* (MRSP) and methicillin-resistant *Staphylococcus aureus* (MRSA) [4]. Similar concerns extend to antifungal resistance in *Malassezia* spp. and dermatophytes [5,6].

The emergence and dissemination of resistant pathogens in companion animals constitute major veterinary and public health concerns. Due to their close and prolonged contact with humans, pets may act as reservoirs and vectors for antimicrobial-resistant microorganisms [7]. The prevalence of MRSP in canine pyoderma has been reported to exceed 35% in some populations, particularly in animals previously exposed to antimicrobial therapy [8]. In addition, household environments and veterinary clinics can serve as important reservoirs for methicillin-resistant staphylococci, including MRSP and MRSA, which often harbor multiple antimicrobial resistance and virulence genes transmissible among animals, humans, and the environment [9,10]. These findings emphasize the epidemiological importance of infection control practices, hygiene measures, and antimicrobial stewardship in veterinary settings. In response to these concerns, the International Society for Companion Animal Infectious Diseases has updated its recommendations to promote culture-guided therapies and prudent antimicrobial use to limit the spread of resistance [11].

These challenges underscore the need to adopt a One Health perspective, which recognizes the dynamic interconnection among animal, human, and environmental health. The increasing population of companion animals in urban households, combined with the global spread of resistant pathogens, emphasizes the need for collaborative surveillance and integrated management strategies [12]. Effective control of infectious dermatitis and associated AMR requires coordinated multidisciplinary efforts to improve diagnostic accuracy, optimize antimicrobial supervision, and reduce the risk of zoonotic transmission. Within this framework, the present study aimed to investigate the presence of *Staphylococcus* species, dermatophytes, and ectoparasites in companion animals presenting and to evaluate the antimicrobial resistance profiles of the isolated *Staphylococcus* strains. By isolating and characterizing these bacterial strains present in pet skin, this study seeks to improve the understanding of their epidemiological distribution and pathogenic potential in veterinary dermatology. Furthermore, assessing their susceptibility to commonly used antibiotics will provide valuable insights into resistance patterns, contributing to more effective therapeutic strategies and supporting efforts to mitigate the spread of antimicrobial resistance in both animal and public health contexts.

## 2. Materials and Methods

### 2.1. Collection and Sample Size

A total of 58 individual animals presenting clinical signs of infectious dermatitis were included in the study between September 2025 and January 2026 in the district of Porto, Portugal. Animals were selected based on the presence of clinical signs consistent with infectious dermatitis at the time of sampling, including skin lesions affecting the face, ears, thorax, abdomen, or limbs; pruritus; alopecia; crust formation; and erythema. Complete medical history and clinical information were collected for each animal. All data were recorded using a structured form, including date of sampling, age, sex, weight, breed, vaccination status, deworming status, encompassing both internal and external antiparasitic treatments. Additional information included lesion description and location, previous dermatological treatments, medical history, and environmental factors. Animals were categorized into three age groups (young: 0–1 year, adult: 1–7 years, and senior: >7 years), according to standardized epidemiological criteria. These classifications were applied uniformly across breeds for descriptive purposes and do not reflect breed-specific differences in physical maturity.

A skin swab was collected from each animal (n = 58) for bacteriological analysis using a sterile swab. When clinically indicated, additional samples were collected for dermatophyte culture (n = 9) using a sterile toothbrush, and for ectoparasite detection (n = 4) by skin scraping with a scalpel blade. These additional procedures were performed only in cases with clinical suspicion of the respective pathogens.

### 2.2. Microbial Isolation and Species Identification

For bacterial isolation, swab samples were plated on Mannitol Salt Agar and incubated at 37 °C for 24 h. For each sample, one to two colonies exhibiting typical *Staphylococcus* morphology were selected and sub-cultured on Brain Heart Infusion (BHI) Agar. Isolates were subsequently stored for further species identification in BHI broth with 15% glycerol at −80 °C. Species identification was performed using matrix-assisted laser desorption ionization time-of-flight mass spectrometry (MALDI-TOF MS) method, with a MALDI Biotyper Sirius system (Bruker Daltonics, Bremen, Germany). Spectra were acquired and analyzed with the manufacturer’s software (Bruker Biotyper, version 5.1.360.35) and compared against the reference database (Bruker Taxonomy Library, version MBT Compass HT IVD Library V2023.0.0.0/MBT Compass HT IVD Library Extension V1.0.0.0). Identification scores were interpreted according to the manufacturer’s criteria, with scores ≥ 2.0 considered reliable for species-level identification, scores between 1.7 and 1.99 for genus-level identification, and scores < 1.7 regarded as unreliable.

For dermatophyte isolation, the samples were inoculated onto selective Dermatophyte Test Medium (DTM) and incubated at 25 °C for 21 days.

For ectoparasite detection, including *Demodex* spp., *Sarcoptes* spp., and *Otodectes* spp., skin scrapings were microscopically examined for identification.

### 2.3. Antimicrobial Susceptibility Testing

Antibiotic susceptibility testing of *Staphylococcus* isolates was performed using the disk diffusion method. A panel of 12 antibiotics was evaluated including oxacillin (1 μg), penicillin (10 U), cefoxitin (30 μg), erythromycin (15 μg), clindamycin (2 μg), linezolid (10 μg), trimethoprim + sulfamethoxazole (1.25 + 23.75 μg), ciprofloxacin (5 μg), rifampicin (5 μg), gentamicin (10 μg), chloramphenicol (30 μg), and tetracycline (30 μg). This panel comprised antibiotics used in both veterinary and human medicine, as well as agents reserved exclusively for human use, highlighting the importance of monitoring antimicrobial resistance across sectors and its relevance to both veterinary care and public health. *Staphylococcus aureus* ATCC 25923 was used as a quality control strain. Bacterial suspensions were prepared in sterile saline solution (0.9% NaCl) and adjusted to a 0.5 McFarland standard. These suspensions were then inoculated onto Mueller–Hinton II agar (Alliance Bio Expertise, Guipry Messac, France) using sterile swabs and the lawn culture method. Antibiotic disks were applied to the agar surface, and the plates were incubated at 37 °C for 18 to 24 h. Following incubation, inhibition zone diameters were measured in millimeters for each isolate and interpreted according to established breakpoints [13,14]. Classification of isolates as susceptible or resistant was based on the Clinical and Laboratory Standards Institute (CLSI) guidelines [13]. At the same time, interpretation for linezolid followed the European Committee on Antimicrobial Susceptibility Testing (EUCAST) guidelines [14].

### 2.4. Analysis of Antibiotic Resistance Genes

The boiling method was used to extract DNA from *Staphylococcus* isolates [15]. Antimicrobial resistance genes were investigated by PCR using the Xpert Fast Hotstart Mastermix (GRiSP Research Solutions, Porto, Portugal), following the manufacturer’s instructions. The targeted genes included common determinants associated with resistance to β-lactams (*mec*A, *mec*C), tetracyclines (*tet*K, *tet*M), ampicillin and penicillin (*bla*Z), trimethoprim (dfrK), macrolides, lincosamides, and streptogramin B (ermT), as well as pleuromutilin, lincosamides, and streptogramin A (*vga*C). The PCR conditions, including primer sequences and annealing temperatures, were described for each primer pair [16,17,18,19,20] and are presented in Table 1.

The nucleotide sequence was amplified using a thermocycler (MJ Mini, Bio-Rad, Hercules, CA, USA) using the following thermal cycling conditions: initial denaturation at 95 °C for 3 min, followed by 40 cycles of denaturation at 95 °C for 15 s, annealing at 45 °C to 59 °C for 15 s, depending on the primer pair (Table 1), and extension at 72 °C for 2 s. A final extension step was carried out at 72 °C for 5 min. PCR assay results were analyzed by electrophoresis on a 1.2% agarose gel, containing 3 μL of GelRed fluorescent dye (GelRed^®^ nucleic acid stain, Biotium, Fremont, CA, USA). A molecular weight marker (NzyDNA Ladder V, Nzytech, Lisbon, Portugal) was included to assist interpretation, and the results were visualized using a transilluminator (ChemiDoc, Bio-Rad, Hercules, CA, USA).

## 3. Results

### 3.1. Distribution of Staphylococci Isolates

**Figure 1:**
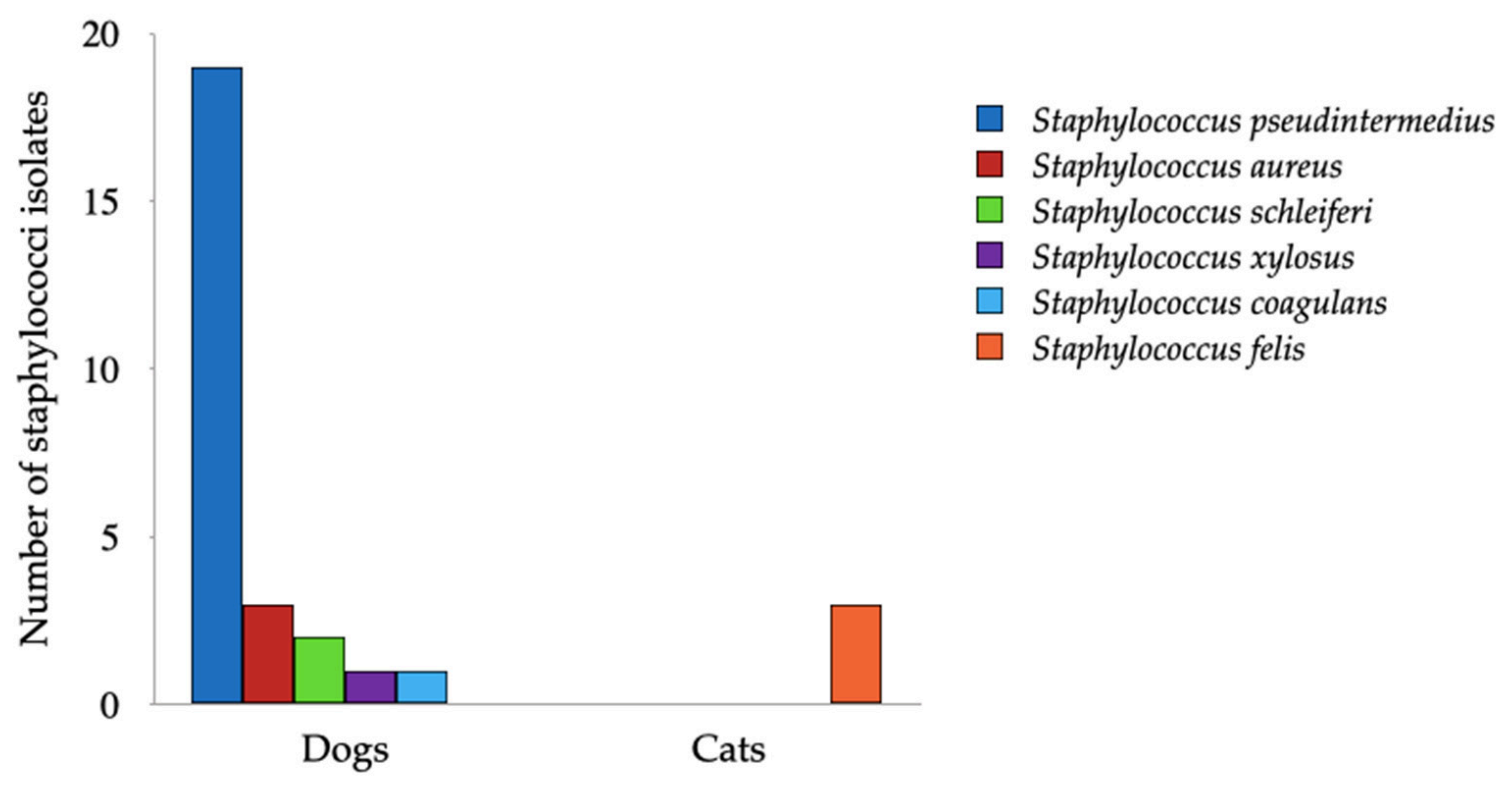
Distribution of *Staphylococcus* species (n = 29) identified in companion animals, including dogs and cats.

A total of 58 cases of infectious dermatitis were analyzed, comprising 58 skin samples collected from 56 dogs and 2 cats for the detection of *Staphylococcus* species. Of the 58 samples, 27 (46.5%) showed no presumptive growth of *Staphylococcus*, while 3 (5.2%) yielded bacterial growth identified as non-*Staphylococcus* species. From the remaining 28 samples, a total of 29 isolates were identified as *Staphylococcus* spp. by MALDI-TOF mass spectrometry (Figure 1). Among the identified staphylococcal isolates, the following species were detected: *Staphylococcus pseudintermedius* (n = 19, 65.6%), *Staphylococcus aureus* (n = 3, 10.4%), *Staphylococcus schleiferi* (n = 2, 6.8%), *Staphylococcus xylosus* (n = 1, 3.4%), and *Staphylococcus coagulans* (n = 1, 3.4%) from dogs. In cats, *Staphylococcus felis* (n = 3, 10.4%) was identified from two animals.

Among canine samples, 26 staphylococci isolates were identified, of which *S. pseudintermedius* (19/26, 73.1%) was the predominant species, followed by *S. aureus* (3/26, 11.5%). In cats, three *S. felis* (3/3, 100%) isolates were identified from two animals, one male and one female, both adults, neutered and of European breed.

Overall, most *Staphylococcus*-positive animals were dogs (90%), adults (83%), male (66%), intact (55%) animals (Table 2). French Bulldogs were the most representative purebreds in this study (28%). Other purebred breeds included Boxer, Dalmatian, German Spitz, Jack Russel Terrier and Labrador Retriever. Vaccination and deworming histories were available for most cases, with the majority of animals reported to be up to date for both preventive measures (Table 2).

At the physical examination, generalized dermatological lesions represented the most common clinical presentation (41%), followed by ear infections (24%), while abdominal, limb, head, and tail lesions were less frequently observed (Table 3). Generalized skin lesions were primarily associated with *S. pseudintermedius*, *S. aureus*, and *S. schleiferi*. *Staphylococcus felis* was more frequently recovered from abdominal lesions, whereas *S. coagulans* was identified in a case presenting with an ear lesion. Among the clinical signs observed, erythema was the most frequent finding, affecting 70% of animals, followed by alopecia (59%) and pruritus (45%).

### 3.2. Distribution of Dermatophytes and Ectoparasite Isolates

No dermatophyte infections were detected. However, two ectoparasite species were identified. *Otodectes cynotis* was observed in an adult male cat presenting with bilateral otitis externa. This animal was also positive for *S. felis*. *Demodex canis* was detected in a 4-month-old female dog presenting with generalized alopecia and erythema.

### 3.3. Phenotypic Antimicrobial Resistance

**Figure 2:**
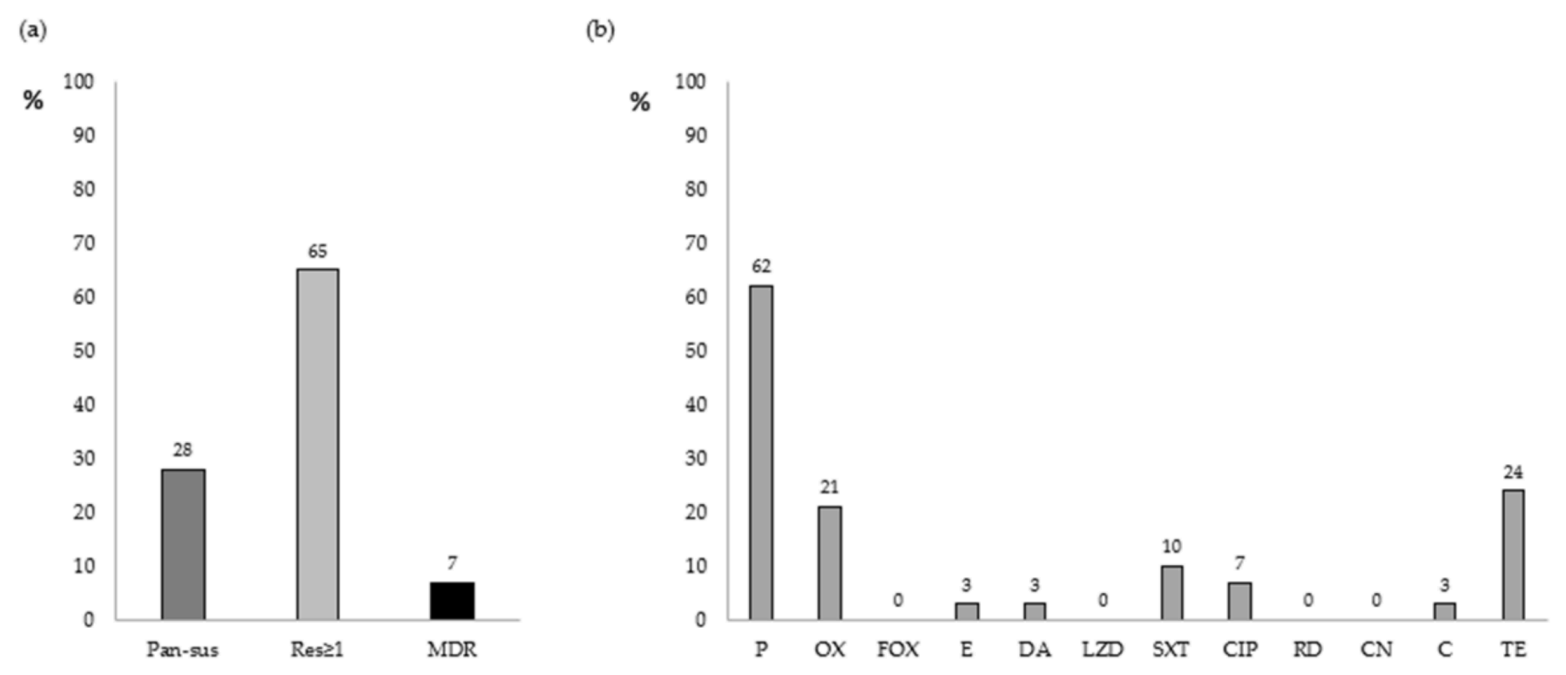
Phenotypic antimicrobial resistance in *Staphylococcus* isolates from companion animals, presented as percentages: (**a**) antibiotic susceptibility profiles; and (**b**) antibiotic resistance profiles. Pan-sus: pan-susceptibility; Res ≥ 1: resistance to ≥1 antibiotic; MDR: multidrug-resistant phenotype; P: penicillin; OX: oxacillin; FOX: cefoxitin; E: erythromycin; DA: clindamycin; LZD: linezolid; SXT: trimethoprim + sulfamethoxazole; CIP: ciprofloxacin; RD: rifampicin; CN: gentamicin; C: chloramphenicol; TE: tetracycline.

All obtained *Staphylococcus* isolates were subjected to antimicrobial susceptibility testing (Table S1). Most isolates (65%) exhibited resistance to at least one antimicrobial agent, whereas 28% of isolates were susceptible to all tested antibiotics, called pan-susceptible. However, multidrug-resistant isolates accounted for 7% of all isolates and were exclusively identified among *S. pseudintermedius* strains (Figure 2a). One isolate exhibited resistance to penicillin, trimethoprim-sulfamethoxazole, and tetracycline (P–SXT–TE), whereas the other showed resistance to penicillin, oxacillin, erythromycin, clindamycin, chloramphenicol, and tetracycline (P–OX–E–DA–C–TE).

Penicillin resistance was the most frequently observed phenotype, affecting 62% of resistant isolates, followed by tetracycline (24%) and oxacillin (21%) resistance. Lower resistance frequencies were observed for trimethoprim-sulfamethoxazole (10%) and ciprofloxacin (7%), whereas resistance to erythromycin, clindamycin, and chloramphenicol was detected in only 3% of isolates each. No resistance was detected to cefoxitin, linezolid, rifampicin, or gentamicin (Figure 2b).

No phenotypic antibiotic resistance profile was detected among the feline isolates. All *S. felis* isolates were pan-susceptible to all antibiotics tested, indicating that resistance in the present study was exclusively associated with canine isolates.

**Figure 3:**
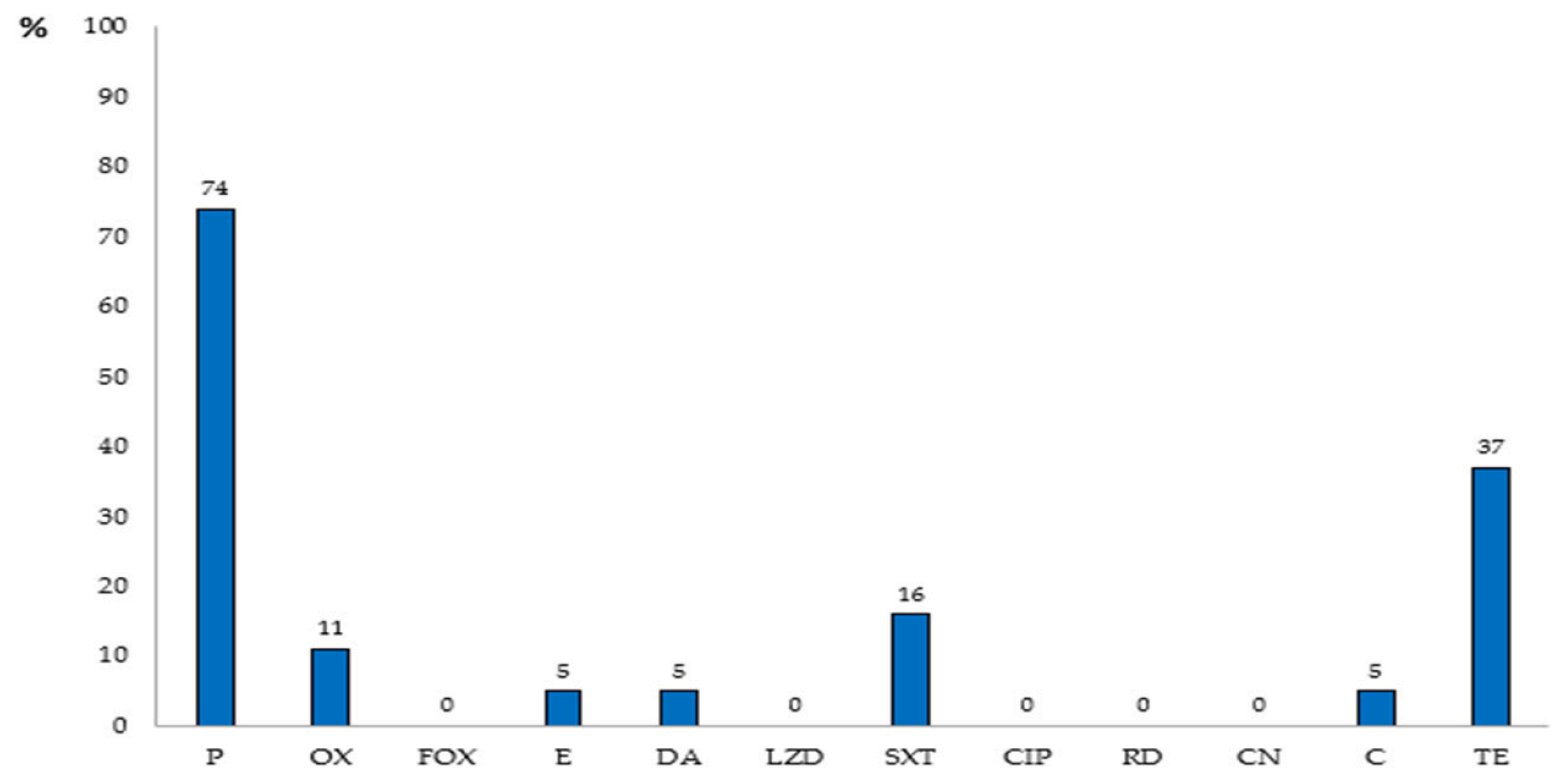
Antimicrobial resistance profiles of *Staphylococcus pseudintermedius* isolates from dogs (n = 19), expressed as percentages. P: penicillin; OX: oxacillin; FOX: cefoxitin; E: erythromycin; DA: clindamycin; LZD: linezolid; SXT: trimethoprim + sulfamethoxazole; CIP: ciprofloxacin; RD: rifampicin; CN: gentamicin; C: chloramphenicol; TE: tetracycline.

Among canine staphylococcal isolates, *S. pseudintermedius* was the most prevalent species and accounted for the majority of resistant isolates. Within this species, resistance to penicillin was detected in 74% of isolates, followed by tetracycline (37%), trimethoprim-sulfamethoxazole (16%), and oxacillin (11%). Resistance to erythromycin, clindamycin, and chloramphenicol was uncommon (5% each). No resistance was observed to cefoxitin, linezolid, ciprofloxacin, rifampicin, or gentamicin (Figure 3).

*Staphylococcus aureus*, although less frequently identified, also exhibited antibiotic resistance profiles. Resistance to β-lactam antibiotics was detected in one-third of the canine isolates, including resistance to penicillin and oxacillin (33% each). In contrast, the remaining isolates were pan-susceptible to the other antibiotics tested. No multidrug-resistant *S. aureus* isolate was detected.

### 3.4. Antibiotic Resistance Genes

**Figure 4:**
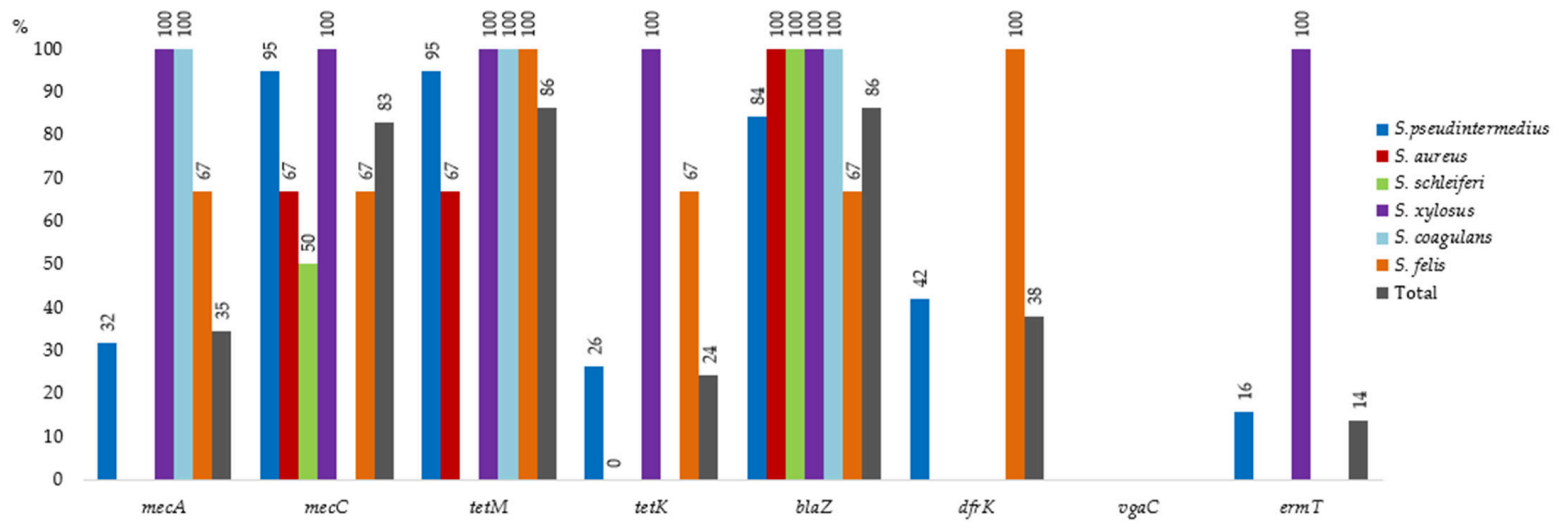
Percentage of antibiotic resistance genes detected in *Staphylococcus* spp. isolates (n = 29).

Genotypic screening for antibiotic resistance was performed in all *Staphylococcus* isolates and compared with their corresponding phenotypic profiles. A high prevalence of antimicrobial resistance genes was observed among the *Staphylococcus* isolates. Overall, the most frequently detected genes were *tet*M and *bla*Z (86% each), followed by *mec*C (83%), *dfr*K (38%), *mec*A (35%), *tet*K (24%), and *erm*T (14%). None of the isolates carried the *vga*C gene (Figure 4).

Among isolates from canine samples, *tet*M and *bla*Z (89% each) were the predominant resistance genes, followed by *mec*C (85%). The distribution of resistance genes varied according to *Staphylococcus* species. In *S. pseudintermedius*, the most prevalent species identified in this study, *mec*C and *tet*M were detected in nearly all isolates (95% each), while *bla*Z (84%) was also highly prevalent (Figure 4). Most *S. pseudintermedius* isolates harbored multiple resistance genes simultaneously, with several isolates carrying combinations of β-lactam resistance genes (*mec*A, *mec*C, and *bla*Z) together with tetracycline resistance determinants (*tet*M and/or *tet*K).

All *S. aureus* isolates carried *bla*Z, while *mec*C and *tet*M were detected in 67% of isolates. Notably, the isolate exhibiting phenotypic resistance to penicillin and oxacillin harbored *tet*M and *bla*Z, whereas the *mec*A gene was not detected in this species (Figure 4).

Among feline isolates, *tet*M and *dfr*K were detected in all isolates. Despite the absence of phenotypic antimicrobial resistance, all feline isolates carried multiple resistance determinants, indicating the presence of resistance genes that were not phenotypically expressed.

Overall, genotypic findings were only partially consistent with phenotypic antimicrobial susceptibility profiles (Table 4). Several isolates carried one or more resistance genes despite exhibiting complete phenotypic susceptibility, particularly among *S. felis*. In contrast, phenotypic resistance to β-lactams and tetracyclines was generally associated with the presence of the corresponding resistance genes, including *bla*Z/*mec*A/*mec*C and *tet*M/*tet*K, respectively. These findings highlight the widespread distribution of antimicrobial resistance genes among staphylococcal species associated with companion animals and emphasize the importance of combining phenotypic and molecular approaches when assessing antimicrobial resistance (Figure S1 and Table S1).

## 4. Discussion

This study provides further insight into infectious dermatitis in companion animals, dogs and cats. It highlights the importance of antimicrobial resistance surveillance in veterinary dermatology, particularly in Portugal, where epidemiological data remain relatively limited. Although several Portuguese studies have recently investigated *S. pseudintermedius* and methicillin-resistant staphylococci in companion animals, available information is still limited compared with other European countries. Only a few studies have evaluated antimicrobial resistance patterns in companion animals in Portugal, despite the increasing importance of multidrug-resistant *Staphylococcus* spp. in veterinary medicine [21]. The limited epidemiological knowledge regarding *S. pseudintermedius* skin and soft tissue infections in Portugal highlights the need for continued surveillance programs [22]. Therefore, the present work provides relevant regional data and reinforces the importance of monitoring infectious skin diseases in dogs and cats within the Portuguese veterinary context.

In this study, *S. pseudintermedius* represented 65.6% of all staphylococcal isolates and 73.1% of canine isolates, confirming its predominance among companion animal skin infections. These findings are consistent with other Portuguese studies that reported the predominance of *S. pseudintermedius* in skin infections and confirmed its major role as the principal bacterial pathogen associated with canine pyoderma and other skin and soft tissue infections [21,22]. *Staphylococcus pseudintermedius* is responsible for up to 92% of canine pyoderma cases and remains one of the main opportunistic pathogens in companion animals [22].

Although *S. aureus* was recovered less frequently in the present study, it remains a clinically relevant pathogen in companion animals due to its zoonotic potential and its association with skin and soft tissue infections. In our study, *S. aureus* accounted for 10.4% of all *Staphylococcus* isolates. Similar observations have been reported in Portugal, where *S. aureus* represented approximately 10% of staphylococcal isolates recovered from companion animals with skin and soft tissue infections in Lisbon, confirming that this species is an occasional but important cause of dermatological infections in pets [23]. In this study, 33% of *S. aureus* isolates exhibited resistance to both penicillin and oxacillin, highlighting the need for continued surveillance of methicillin-resistant staphylococci in veterinary medicine. Given the potential for transmission of *S. aureus* between humans and companion animals, the detection of this species in canine skin infections highlights the importance of continued surveillance from a One Health perspective [24].

In this study, the detection of *S. felis* exclusively in cats is consistent with its recognized role as a feline-associated staphylococcal species. Previous studies have identified *S. felis* as one of the most common coagulase-negative staphylococci colonizing feline skin and mucosal surfaces [25]. Although all *S. felis* isolates in the present study were phenotypically susceptible to the tested antimicrobials, they carried multiple resistance genes, including *tet*M, *mec*A, *mec*C, *bla*Z, *tet*K, and *dfr*K. This finding suggests that feline-associated staphylococci may constitute an underrecognized reservoir of antimicrobial resistance determinants despite the absence of phenotypic resistance.

Despite providing novel data on *Staphylococcus* spp. associated with infectious dermatitis in companion animals in Portugal, this study has several limitations. One limitation is that the isolation of *Staphylococcus* spp. from skin lesions does not necessarily demonstrate a primary pathogenic role, as several staphylococcal species, such as *S. felis*, are recognized members of the normal skin microbiota of companion animals and may behave as opportunistic pathogens [26]. Nevertheless, all samples included in this study were collected exclusively from animals presenting clinical signs of infectious dermatitis, and bacteriological analyses were performed only when the attending veterinarian clinically suspected a bacterial infection. Therefore, the isolates were considered clinically relevant in the context of the observed lesions. However, bacterial culture alone cannot definitively distinguish colonization from infection, and the microbiological findings should always be interpreted together with the clinical presentation. Another limitation of this study is that only one to two colonies with typical *Staphylococcus* morphology were selected from each culture plate for further characterization. Although this approach reflects routine diagnostic practice and is intended to identify the predominant isolate, it may have underestimated mixed bacterial infections or the coexistence of multiple *Staphylococcus* species within the same lesion. Moreover, the relatively small sample size, particularly the inclusion of two cats, limits the generalization of the findings and prevents meaningful comparisons between dogs and cats. In addition, sampling was restricted to a single geographic region, and therefore the results may not be representative of the epidemiology of *Staphylococcus* spp. in other regions of Portugal. Finally, due to the limited sample size and the descriptive objectives of the study, no statistical analyses were performed.

Antimicrobial resistance remains one of the most concerning findings associated with infectious dermatitis in companion animals. MRSP is described as an increasingly important pathogen in canine pyoderma, particularly due to its multidrug-resistant phenotype and limited therapeutic options [27]. The emergence of methicillin-resistant and multidrug-resistant isolates represents a major challenge in clinical dermatology, as treatment failure is becoming more frequent, especially in recurrent or chronic infections previously exposed to antimicrobial therapy. One Portuguese study reported that 95.8% of MRSP isolates exhibited multidrug resistance profiles, with resistance commonly observed against β-lactams, tetracyclines, macrolides, lincosamides, aminoglycosides, and fluoroquinolones [22]. This finding does not corroborate the results of the present study, in which 65% of *Staphylococcus* isolates exhibited resistance to at least one antimicrobial agent, and 7% of isolates demonstrated multidrug-resistant profiles. Resistance was most frequently observed against penicillin (62%), followed by tetracycline (24%) and oxacillin (21%). The lower MDR prevalence observed in the present study may reflect differences in sample size, geographical distribution, antimicrobial exposure history, or the inclusion of both resistant and susceptible isolates rather than exclusively MRSP strains. Nevertheless, the relatively high frequency of resistance to β-lactam antibiotics remains concerning because these drugs constitute first-line treatments for many dermatological infections in companion animals [11].

It was found that MRSP isolates recovered from canine pyoderma were multidrug-resistant and frequently carried resistance genes, such as *bla*Z, *erm*B, *tet*M, *tet*K, and *dfr*G [27]. The molecular analysis in the present study revealed a widespread distribution of antimicrobial resistance determinants. The most prevalent genes identified were *tet*M, *bla*Z, and *mecC*. These findings support the growing concern that empirical and repeated antimicrobial therapies contribute significantly to the selection and dissemination of resistant isolates in veterinary practice. Within *S. pseudintermedius*, *mec*C and *tet*M were detected in 94.7% of isolates, while *bla*Z was identified in 84.2%. The frequent detection of these genes corresponds well with the phenotypic resistance observed against β-lactams and tetracyclines. Interestingly, several isolates carried resistance determinants despite remaining phenotypically susceptible. This discrepancy was particularly evident among *S. felis* isolates, all of which were phenotypically susceptible but harbored multiple resistance genes, including *tet*M, *bla*Z, *mec*A, *mec*C, *tet*K, and *dfr*K. Similar phenotype-genotype discrepancies have previously been reported in staphylococci. They may be explained by heterogeneous or inducible expression of resistance genes, regulatory mechanisms affecting gene expression, mutations leading to altered gene functionality, or environmental factors influencing phenotypic resistance expression [28]. These findings emphasize the importance of combining phenotypic susceptibility testing and genotypic characterization when evaluating antimicrobial resistance.

Oxacillin resistance was observed in 21% of isolates, while no cefoxitin resistance was detected. In contrast, the high prevalence of *mec*C (83%) and *mec*A (35%) genes was identified. These genes encode the low-affinity penicillin-binding proteins PBP2a and PBP2c, which are typically associated with resistance to β-lactam antibiotics [29,30]. The low levels of phenotypic resistance observed, particularly the absence of cefoxitin resistance, indicate a discrepancy between genotypic and phenotypic findings. This may be explained by reduced or heterogeneous expression of *mec* genes, the presence of non-functional variants, or regulatory mechanisms that limit gene expression under the conditions tested [31]. In addition, *mec*C-mediated resistance has been reported to produce borderline or even susceptible phenotypes under standard testing conditions [29,30], and *mec*C-positive isolates often exhibit lower β-lactam minimum inhibitory concentration than *mec*A-positive strains, which may hinder detection by routine phenotypic methods such as disk diffusion [32,33]. Although the high prevalence of *mec*C detected in this study is noteworthy, future molecular confirmation of representative *mec*C-positive amplicons, such as by Sanger sequencing, would provide additional support for these findings and further strengthen their interpretation. These findings highlight the importance of combining molecular and phenotypic approaches for accurate detection and interpretation of methicillin resistance in *Staphylococcus* spp. The antibiotics most prescribed for canine and feline dermatological infections include β-lactams, such as amoxicillin–clavulanic acid and cephalosporins, as well as fluoroquinolones, macrolides, lincosamides, and tetracyclines [11]. β-lactam antibiotics remain widely used in veterinary medicine for the treatment of *Staphylococcus*-associated infections [1,21]. Pyoderma is one of the primary reasons for antimicrobial prescription in companion animals, and increasing resistance has led to the frequent use of critically important antimicrobials shared between human and veterinary medicine [22]. The widespread administration of these drugs may explain the increasing prevalence of methicillin-resistant and multidrug-resistant staphylococci reported worldwide.

From a One Health perspective, the zoonotic implications of resistant *Staphylococcus* spp. are particularly important. Pets live in close contact with humans and may act as reservoirs and dissemination sources of antimicrobial-resistant bacteria [7,12]. Multidrug-resistant *Staphylococcus* spp. in companion animals is a significant public health concern because resistant strains and resistance genes may be transmitted between animals and humans [21]. *Staphylococcus pseudintermedius* is now recognized as a potential public health issue due to its ability to carry and disseminate antimicrobial resistance determinants [22]. The increasing occurrence of MRSP and MRSA in veterinary settings reinforces the importance of antimicrobial stewardship, hygiene measures, and integrated surveillance programs involving veterinarians, microbiologists, physicians, and public health authorities.

Although dermatophytes are traditionally considered important etiological agents of infectious dermatitis in companion animals, no dermatophytes were identified in the present study. A recent study has reported a global increase in dermatophytosis in Portugal but a decrease in prevalence rates in the North region of Portugal, especially in animals receiving regular veterinary care and preventive treatments [34]. However, other epidemiological studies have described an increasing prevalence of dermatophytosis in dogs and cats worldwide, particularly in regions with higher humidity, environmental contamination, and larger populations of stray or shelter animals [35]. These findings are not consistent with the absence of dermatophyte isolation observed in the present study. Nevertheless, several factors may explain this difference. Climatic conditions in the North of Portugal, improved hygiene practices, and routine veterinary follow-up may contribute to reducing dermatophyte transmission and persistence in household companion animals.

A similarly low prevalence was observed for ectoparasitic agents, with only two ectoparasite-positive cases identified. These results may be explained by the high proportion of animals with a history of previous ectoparasite treatments (79%), most of which had been administered within the previous 3 months (52%). This finding is consistent with recent epidemiological trends showing a decline in ectoparasitic skin diseases in companion animals, due to the widespread use of modern antiparasitic prophylaxis, particularly in countries with high veterinary care coverage and regular prophylactic treatment practices [36]. Advances in preventive veterinary medicine, owner awareness, and improved access to antiparasitic products may therefore explain the limited detection of parasites in the present study.

Overall, these findings reinforce the importance of accurate microbiological diagnosis and antimicrobial susceptibility testing in veterinary medicine, particularly in dermatology. Given the increasing prevalence of resistant *Staphylococcus* spp. and the zoonotic implications associated with antimicrobial resistance, empirical therapy alone should be avoided whenever possible. Instead, culture-guided treatment and responsible antimicrobial use should be prioritized to improve therapeutic success and reduce the selection of multidrug-resistant pathogens in companion animals. Such measures may also contribute to broader antimicrobial stewardship efforts within a One Health framework, although transmission between animals, humans, and the environment was not investigated in the present study.

## 5. Conclusions

This study contributes to the current understanding of bacterial agents present in infectious dermatitis and antimicrobial resistance in companion animals in Portugal, a field in which epidemiological data remain relatively limited. The identification of antimicrobial-resistant isolates further emphasizes that resistant bacteria continue to circulate in companion animals and represent an important challenge for veterinary dermatology and public health. Beyond their clinical relevance, these findings highlight the importance of antimicrobial resistance in companion animals within a One Health context, given the close contact between pets and humans. However, the present study did not assess human or environmental reservoirs, nor did it investigate transmission pathways. The emergence of multidrug-resistant staphylococci highlights the necessity of accurate diagnosis, culture-guided therapy, prudent antimicrobial use, and continuous epidemiological surveillance in veterinary practice.

Overall, the present study reinforces the importance of integrated strategies based on the One Health concept, recognizing the dynamic link between animal, human, and environmental health. Improved surveillance of infectious dermatitis and antimicrobial resistance in companion animals is essential for the development of effective therapeutic and preventive strategies, the preservation of animal welfare, and the mitigation of antimicrobial resistance dissemination. Continued monitoring of dermatological pathogens in pets will contribute to a better understanding of their epidemiology and support more sustainable approaches to veterinary healthcare and public health protection.

## Figures and Tables

**Table 1:** Nucleotide sequences with annealing temperature (AT) and expected amplicon size, used in this study.

Primer	Nucleotide Sequence (5′ to 3′)	Size (bp)	AT (°C)	Reference
*mec*A	CCACTTCATATCTTGAACG TCCAGATTACAACTTCACCAGG	162	59	Larsen et al. [16]
*mec*C	GCTCCTAATGCTAATGCA TAAGCAATAATGACTACC	304	51	Cuny et al. [17]
*tet*K	GTAGCGACAATAGGTAATAGT GTAGTGACAATAAACCTCCTA	360	58	Meroni et al. [18]
*tet*M	AGTGGAGCGATTACAGAA CATATGTCCTGGCGTGTCTA	158	55	Meroni et al. [18]
*bla*Z	ACTTCAACACCTGCTGCTTTC TGACCACTTTTATCAGCAACC	173	52	Argudín et al. [19]
*dfr*K	GCTGCGATGGATAATGAACAG GGACGATTTCACAACCATTAAAGC	214	50	Fessler et al. [20]
*erm*T	ATTGGTTCAGGGAAAGGTCA GCTTGATAAAATTGGTTTTTGG	536	45	Fessler et al. [20]
*vga*C	CCGTATGCCCAGAGTGAGAT TGCTTGGGAACAAGTCCTTC	671	58	Fessler et al. [20]

**Table 2:** Characteristics of *Staphylococcus* dermatitis in dogs and cats.

General Information		*S. pseudintermedius* (n = 19)	*S. aureus* (n = 3)	*S. schleiferi* (n = 2)	*S. xylosus* (n = 1)	*S. coagulans* (n = 1)	*S. felis* (n = 3)	Total (n = 29)
**Species**	Dog	19 (100%)	3 (100%)	2 (100%)	1 (100%)	1 (100%)	0 (0%)	26 (89.7%)
	Cat	0 (0%)	0 (0%)	0 (0%)	0 (0%)	0 (0%)	3 (100%)	3 (10.3%)
**Sex**	Male	12 (63.2%)	3 (100%)	2 (100%)	0 (0%)	1 (100%)	1 (33.3%)	19 (65.5%)
	Female	7 (36.8%)	0 (0%)	0 (0%)	1 (100%)	0 (0%)	2 (66.7%)	10 (34.5%)
**Age** (years)	Young (0–1)	1 (5.3%)	0 (0%)	0 (0%)	1 (100%)	0 (0%)	0 (0%)	2 (6.9%)
	Adult (1–7)	15 (78.9%)	3 (100%)	2 (100%)	0 (0%)	1 (100%)	3 (100%)	24 (82.8%)
	Senior (>7)	3 (15.8%)	0 (0%)	0 (0%)	0 (0%)	0 (0%)	0 (0%)	3 (10.3%)
**Reproductive status**	Intact	14 (73.7%)	0 (0%)	0 (0%)	1 (100%)	1 (100%)	0 (0%)	16 (55.2%)
	Neutered	5 (26.3%)	1 (33.3%)	0 (0%)	0 (0%)	0 (0%)	3 (100%)	9 (31%)
	Unknown	0 (0%)	2 (66.7%)	2 (100%)	0 (0%)	0 (0%)	0 (0%)	4 (13.8%)
**Breed**	French Bulldog	6 (31.6%)	2 (66.7%)	0 (0%)	0 (0%)	0 (0%)	0 (0%)	8 (27.6%)
	Other purebred	8 (42.1%)	0 (0%)	0 (0%)	1 (100%)	1 (100%)	0 (0%)	10 (34.5%)
	Mixed	5 (26.3%)	1 (33.3%)	2 (100%)	0 (0%)	0 (0%)	0 (0%)	8 (27.6%)
	European cat	0 (0%)	0 (0%)	0 (0%)	0 (0%)	0 (0%)	3 (100%)	3 (10.3%)
**Body weight**	<5 kg	0 (0%)	1 (33.3%)	0 (0%)	0 (0%)	0 (0%)	1 (33.3%)	2 (6.9%)
	5–14 kg	11 (57.9%)	0 (0%)	0 (0%)	1 (100%)	1 (100%)	2 (66.7%)	15 (51.7%)
	15–30 kg	5 (26.3%)	0 (0%)	0 (0%)	0 (0%)	0 (0%)	0 (0%)	5 (17.2%)
	>30 kg	2 (10.5%)	0 (0%)	0 (0%)	0 (0%)	0 (0%)	0 (0%)	2 (6.9%)
	Unknown	1 (5.3%)	2 (66.7%)	2 (100%)	0 (0%)	0 (0%)	0 (0%)	5 (17.2%)
**Vaccination status**	Vaccinated	14 (73.7%)	0 (0%)	0 (0%)	1 (100%)	1 (100%)	2 (66.7%)	18 (62.1%)
	Unvaccinated	3 (15.8%)	1 (33.3%)	0 (0%)	0 (0%)	0 (0%)	1 (33.3%)	5 (17.2%)
	Unknown	2 (10.5%)	2 (66.7%)	2 (100%)	0 (0%)	0 (0%)	0 (0%)	6 (20.7%)
**Deworming status**	Treated	11 (57.9%)	0 (0%)	0 (0%)	1 (100%)	1 (100%)	2 (66.7%)	15 (51.7%)
	Non-treated	6 (31.6%)	1 (33.3%)	0 (0%)	0 (0%)	0 (0%)	1 (33.3%)	8 (27.6%)
	Unknown	2 (10.5%)	2 (66.7%)	2 (100%)	0 (0%)	0 (0%)	0 (0%)	6 (20.7%)
**Outdoor** **access**	No	17 (89.5%)	3 (100%)	2 (100%)	1 (100%)	1 (100%)	0 (0%)	24 (82.8%)
	Yes	2 (10.5%)	0 (0%)	0 (0%)	0 (0%)	0 (0%)	3 (100%)	5 (17.2%)
	Unknown	0 (0%)	0 (0%)	0 (0%)	0 (0%)	0 (0%)	0 (0%)	0 (0%)
**Housing conditions**	Single-animal	15 (78.9%)	0 (0%)	0 (0%)	1 (100%)	1 (100%)	1 (33.3%)	18 (62.1%)
	Multi-animals	4 (21.1%)	1 (33.3%)	0 (0%)	0 (0%)	0 (0%)	0 (0%)	5 (17.2%)
	Unknown	0 (0%)	2 (66.7%)	2 (100%)	0 (0%)	0 (0%)	2 (66.7%)	6 (20.7%)

**Table 3:** Clinical characteristics and lesion distribution in companion animals by isolated *Staphylococcus* species.

Clinical Characteristics		*S. pseudintermedius* (n = 19)	*S. aureus* (n = 3)	*S. schleiferi* (n = 2)	*S. xylosus* (n = 1)	*S. coagulans* (n = 1)	*S. felis* (n = 3)	Total (n = 29)
**Location** **of the skin** **lesion**	Head	2 (10.5%)	0 (0%)	0 (0%)	0 (0%)	0 (0%)	0 (0%)	2 (6.9%)
	Ears	5 (26.3%)	0 (0%)	0 (0%)	0 (0%)	1 (100%)	1 (33.3%)	7 (24.2%)
	Thorax	0 (0%)	0 (0%)	0 (0%)	0 (0%)	0 (0%)	0 (0%)	0 (0%)
	Abdomen	1 (5.3%)	0 (0%)	0 (0%)	1 (100%)	0 (0%)	2 (66.7%)	4 (13.8%)
	Limbs	2 (10.5%)	1 (33.3%)	0 (0%)	0 (0%)	0 (0%)	0 (0%)	3 (10.3%)
	Tail	1 (5.3%)	0 (0%)	0 (0%)	0 (0%)	0 (0%)	0 (0%)	1 (3.4%)
	Generalized	8 (42.1%)	2 (66.7%)	2 (100%)	0 (0%)	0 (0%)	0 (0%)	12 (41.4%)
**Pruritus**	Yes	6 (31.6%)	2 (66.7%)	2 (100%)	1 (100%)	0 (0%)	2 (66.7%)	13 (44.8%)
	No	13 (68.4%)	0 (0%)	0 (0%)	0 (0%)	1 (100%)	1 (33.3%)	15 (51.7%)
	Unknown	0 (0%)	1 (33.3%)	0 (0%)	0 (0%)	0 (0%)	0 (0%)	1 (3.4%)
**Alopecia**	Yes	13 (68.4%)	1 (33.3%)	0 (0%)	1 (100%)	0 (0%)	2 (66.7%)	17 (58.6%)
	No	6 (31.6%)	0 (0%)	0 (0%)	0 (0%)	1 (100%)	1 (33.3%)	8 (27.6%)
	Unknown	0 (0%)	2 (66.7%)	2 (100%)	0 (0%)	0 (0%)	0 (0%)	4 (13.8%)
**Erythema**	Yes	14 (73.7%)	1 (33.3%)	0 (0%)	1 (100%)	1 (100%)	3 (100%)	20 (70%)
	No	5 (26.3%)	0 (0%)	0 (0%)	0 (0%)	0 (0%)	0 (0%)	5 (17.2%)
	Unknown	0 (0%)	2 (66.7%)	2 (100%)	0 (0%)	0 (0%)	0 (0%)	4 (13.8%)
**Crusts**	Yes	4 (21%)	0 (0%)	0 (0%)	1 (100%)	1 (100%)	0 (0%)	6 (20.7%)
	No	15 (79%)	1 (33.3%)	0 (0%)	0 (0%)	0 (0%)	3 (100%)	19 (65.5%)
	Unknown	0 (0%)	2 (66.7%)	2 (100%)	0 (0%)	0 (0%)	0 (0%)	4 (13.8%)

**Table 4:** Phenotypic and genotypic characteristics of *Staphylococcus* spp. isolates (n = 29).

*Staphylococcus* Species	Antibiotic Resistance Phenotype ^†^	Antibiotic Resistance Genotype ^‡^
*Staphylococcus pseudintermedius*	TE	*mec*C, *tet*M, *tet*K, *bla*Z
*Staphylococcus pseudintermedius*	P	*mec*C, *tet*M, *bla*Z, *dfr*K
*Staphylococcus pseudintermedius*	P, SXT	*mec*C, *tet*M, *bla*Z, *dfr*K, *erm*T
*Staphylococcus pseudintermedius*	P, OX	*tet*M, *bla*Z, *dfr*K
*Staphylococcus pseudintermedius*	P, TE	*mec*C, *tet*M, *bla*Z
*Staphylococcus pseudintermedius* *	P, SXT, TE	*mec*A, *mec*C, *tet*M, *tet*K, *bla*Z, *dfr*K
*Staphylococcus pseudintermedius*	Negative	*mec*C, *tet*M
*Staphylococcus pseudintermedius* *	P, OX, E, DA, C, TE	*mec*A, *mec*C, *tet*M, *bla*Z, *dfr*K, *erm*T
*Staphylococcus pseudintermedius*	Negative	*mec*A, *mec*C, *tet*M, *bla*Z
*Staphylococcus pseudintermedius*	P	*mec*A, *mec*C, *tet*M, *bla*Z
*Staphylococcus pseudintermedius*	TE	*mec*C, *tet*M, *bla*Z
*Staphylococcus pseudintermedius*	P	*mec*C
*Staphylococcus pseudintermedius*	TE	*mec*C, *tet*M, *bla*Z
*Staphylococcus pseudintermedius*	P	*mec*C, *tet*M, *bla*Z
*Staphylococcus pseudintermedius*	P	*mec*C, *tet*M, *bla*Z
*Staphylococcus pseudintermedius*	P	*mec*A, *mec*C, *tet*M, *dfr*K
*Staphylococcus pseudintermedius*	P, TE	*mec*C, *tet*M, *tet*K, *bla*Z
*Staphylococcus pseudintermedius*	P, SXT	*mec*C, *tet*M, *tet*K, *bla*Z, *dfr*K, *erm*T
*Staphylococcus pseudintermedius*	P	*mec*A, *mec*C, *tet*M, *tet*K, *bla*Z, *dfr*K
*Staphylococcus aureus*	Negative	*mec*C, *tet*M, *bla*Z
*Staphylococcus aureus*	P, OX	*tet*M, *bla*Z
*Staphylococcus aureus*	Negative	*mec*C, *bla*Z
*Staphylococcus schleiferi*	P, OX, CIP	*bla*Z
*Staphylococcus schleiferi*	P, OX, CIP	*mec*C, *bla*Z
*Staphylococcus xylosus*	P, OX	*mec*A, *mec*C, *tet*M, *bla*Z, *erm*T
*Staphylococcus coagulans*	Negative	*mec*A, *tet*M, *bla*Z
*Staphylococcus felis*	Negative	*mec*A, *mec*C, *tet*M, *tet*K, *bla*Z, *dfr*K
*Staphylococcus felis*	Negative	*mec*A, *mec*C, *tet*M, *bla*Z, *dfr*K
*Staphylococcus felis*	Negative	*tet*M, *tet*K, *dfr*K

^†^: Antibiotic resistance classes, β-lactams/penicillin (P: penicillin; OX: oxacillin); macrolides (E: erythromycin); lincosamides (DA: clindamycin); folate pathway antagonists (SXT: trimethoprim + sulfamethoxazole); fluoroquinolones (CIP: ciprofloxacin); phenicols (C: chloramphenicol); tetracyclines (TE: tetracycline). ^‡^: antibiotic resistance genes to β-lactams (*mec*A, *mec*C), tetracyclines (*tet*K, *tet*M), ampicillin and penicillin (*bla*Z), trimethoprim (*dfr*K), and macrolides, lincosamides and streptogramin B (*erm*T). *: multidrug-resistant (MDR) isolates.

## Data Availability

Data is contained within the article. The data will be made available upon request to the authors.

## Supplementary Materials

The following supporting information can be downloaded at https://www.mdpi.com/article/10.3390/ani16152284/s1: Table S1. Disk diffusion inhibition zone diameters (mm) for all *Staphylococcus* isolates included in the study. Figure S1. Representative agarose gel electrophoresis of PCR amplification products for antimicrobial resistance genes detected in *Staphylococcus* spp. isolates. (a) *mec*A and (b) *mec*C.

## Abbreviations

The following abbreviations are used in this manuscript:

AMR	Antimicrobial resistance
MDR	Multidrug resistance
MRSA	Methicillin-resistant *Staphylococcus aureus*
MRSP	Methicillin-resistant *Staphylococcus pseudintermedius*
