# Supplementary material for: Infectious Dermatitis in Companion Animals: A One Health Perspective
Source: Animals (Basel). 2026 Jul 23;16(15):2284. doi: 10.3390/ani16152284 (PMC13463489; doi:10.3390/ani16152284)
Supplement: Supplementary file 1 [file animals-16-02284-s001.zip › animals-4400697-supplementary.pdf]

animals

Supplementary Materials

Infectious Dermatitis in Companion Animals: A One Health Perspective

Lola Cellier [1], Helena Guimarães [1], Ana Cardoso [1], Carla Campos [2,3], Patrícia Barradas [1,4,5] and Carla Miranda [1,4,5,6,*]

1 University Institute of Health Sciences, Cooperativa de Ensino Superior Politécnico e Universitário (IUCS-CESPU), Avenida Central de Gandra 1317, 4585-116 Paredes, Portugal; a30486@alunos.cespu.pt (L.C.); helena.guimaraes@iucs.cespu.pt (H.G.); ana.cardoso@cespu.pt (A.C.); patricia.barradas@iucs.cespu.pt (P.B.)
2 Instituto Português de Oncologia do Porto Francisco Gentil, Rua Dr. António Bernardino de Almeida, 4200-072 Porto, Portugal; carla.campos@ipoporto.min-saude.pt
3 Escola Superior de Saúde, Instituto Politécnico do Porto, Rua Dr. António Bernardino de Almeida, 4200-072 Porto, Portugal
4 UCIBIO—Applied Molecular Biosciences Unit, University Institute of Health Sciences (1H-TOXRUN, IUCS-CESPU), Avenida Central de Gandra 1317, 4585-116 Paredes, Portugal
5 Associate Laboratory i4HB—Institute of Health and Bioeconomy, University Institute of Health Sciences, CESPU, Avenida Central de Gandra 1317, 4585-116 Paredes, Portugal
6 LAQV-REQUIMTE—Associated Laboratory for Green Chemistry, University NOVA of Lisbon, 1099-085 Caparica, Portugal
* Correspondence: carla.miranda@iucs.cespu.pt

 https://doi.org/10.3390/ani16152284

Table S1. Disk diffusion inhibition zone diameters (mm) for all *Staphylococcus* isolates included in the study.

***Staphylococcus* species**	**P 10U**	**Ox 1μg**	**FOX 30μg**	**E 15μg**	**DA 2μg**	**LZD 10μg**	**SXT 1.25+23.75**	**CIP 5μg**	**RD 5μg**	**CN 10μg**	**C 30μg**	**TE 30μg**	**Antibiotic Resistance Phenotype**
S. pseudintermedius	46	22	40	32	33	36	17	34	36	22	32	14	TE
S. pseudintermedius	21	18	34	30	26	28	22	28	34	24	30	24	P
S. pseudintermedius	24	22	42	34	32	36	0	38	40	21	32	36	P, SXT
S. pseudintermedius	27	0	26	28	32	26	30	30	32	22	28	30	P, OX
S. pseudintermedius	22	21	40	34	30	32	21	30	40	25	34	14	P, TE
S. pseudintermedius	22	20	38	34	30	30	0	36	40	24	34	10	P, SXT, TE
S. pseudintermedius	52	24	44	44	36	40	18	36	42	26	32	40	Negative
S. pseudintermedius	17	15	40	0	0	30	22	40	30	15	0	10	P, OX, E, DA, C, TE
S. pseudintermedius	46	22	40	40	34	38	28	34	44	25	34	38	Negative
S. pseudintermedius	23	20	40	34	32	32	17	30	40	26	34	16	P
S. pseudintermedius	48	21	36	40	30	32	26	26	40	21	30	14	TE
S. pseudintermedius	25	21	42	34	32	34	24	30	40	25	32	40	P
S. pseudintermedius	30	22	46	50	32	34	24	34	42	27	34	12	TE
S. pseudintermedius	23	20	40	36	30	32	14	32	42	25	34	40	P
S. pseudintermedius	22	24	42	36	30	34	28	34	40	25	34	40	P
S. pseudintermedius	26	22	46	38	34	36	26	32	42	28	34	40	P
S. pseudintermedius	22	18	36	34	32	30	16	30	36	24	30	14	P, TE
S. pseudintermedius	22	20	44	34	32	34	0	36	40	25	30	40	P, SXT
S. pseudintermedius	26	21	44	38	34	34	14	26	38	26	30	40	P
S. aureus	14	15	28	26	24	24	26	34	30	18	24	30	P, OX
S. aureus	50	25	32	30	30	28	32	34	34	20	28	32	Negative
S. aureus	44	18	30	30	29	28	22	28	36	18	26	32	Negative
S. schleiferi	19	15	36	32	28	30	21	8	36	23	30	36	P, OX, CIP
S. schleiferi	19	17	38	32	30	30	22	10	36	23	30	36	P, OX, CIP
S. xylosus	25	1	30	30	28	28	26	30	26	25	30	34	P, OX
S. coagulans	48	20	32	30	26	30	18	30	30	23	28	32	Negative
S. felis	40	21	36	28	30	28	22	36	36	22	26	30	Negative
S. felis	40	22	38	28	30	32	24	36	34	23	30	36	Negative
S. felis	40	24	40	32	32	34	26	36	38	25	30	38	Negative
S. aureus ATCC 2593	33	23	28	26	27	24	24	24	30	19	24	28	

P: penicillin; OX: oxacillin; FOX: cefoxitin; E: erythromycin; DA: clindamycin; LZD: linezolid; SXT: trimethoprim + sulfamethoxazole; CIP: ciprofloxacin; RD: rifampicin; CN: gentamicin; C: chloramphenicol; TE: tetracycline.

https://doi.org/10.3390/ani16152284

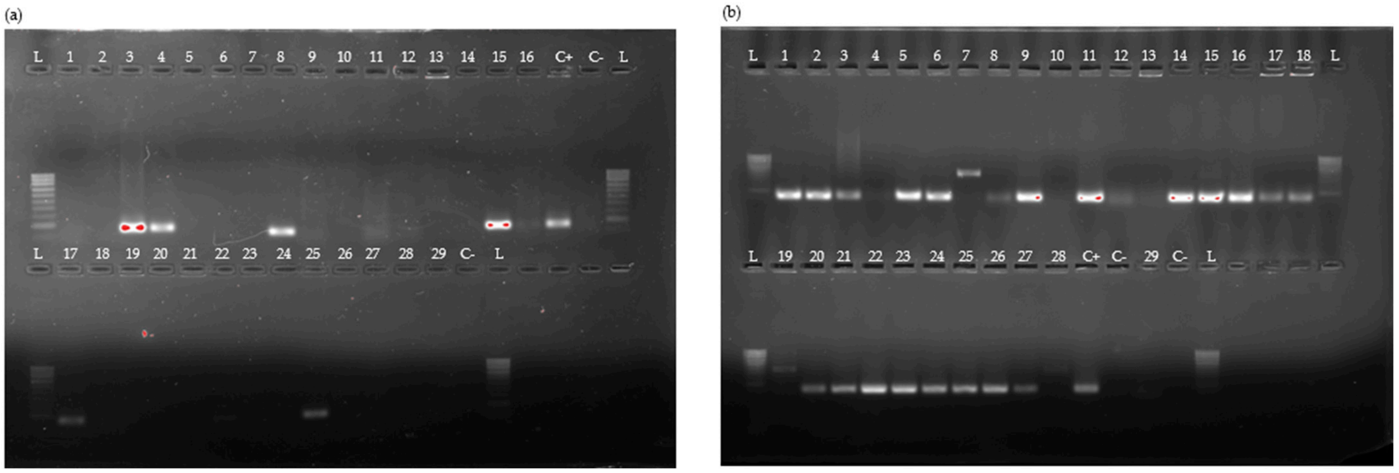

Figure S1. Representative agarose gel electrophoresis of PCR amplification products for antimicrobial resistance genes detected in *Staphylococcus* spp. isolates. (a) *mec*A and (b) *mec*C. L: molecular weight marker (100-1000 bp); 1-31: isolates; C+: positive control; C-: negative control.
